# Role of the membrane-spanning 4A gene family in lung adenocarcinoma

**DOI:** 10.3389/fgene.2023.1162787

**Published:** 2023-07-18

**Authors:** Zijun Zheng, Huiping Li, Runjiao Yang, Hui Guo

**Affiliations:** Department of Endocrinnology, Lequn Branch, The First Hospital of Jilin University, Changchun, China

**Keywords:** lung adenocarcinoma, MS4A gene family, prognosis, gene mutation, tumor immune microenvironment

## Abstract

Lung adenocarcinoma, which is the second most prevalent cancer in the world, has a poor prognosis and a low 5-year survival rate. The MS4A protein family is crucial to disease development and progression, particularly for cancers, allergies, metabolic disorders, autoimmune diseases, infections, and neurodegenerative disorders. However, its involvement in lung adenocarcinoma remains unclear. In this study, we found that 11 MS4A family genes were upregulated or downregulated in lung adenocarcinoma. Furthermore, we described the genetic variation landscape of the MS4A family in lung adenocarcinoma. Notably, through functional enrichment analysis, we discovered that the MS4A family is involved in the immune response regulatory signaling pathway and the immune response regulatory cell surface receptor signaling pathway. According to the Kaplan–Meier curve, patients with lung adenocarcinoma having poor expression of MS4A2, MS4A7, MS4A14, and MS4A15 had a low overall survival rate. These four prognostic genes are substantially associated with immune-infiltrating cells, and a prognosis model incorporating them may more accurately predict the overall survival rate of patients with lung adenocarcinoma than current models. The findings of this study may offer creative suggestions and recommendations for the identification and management of lung adenocarcinoma.

## Introduction

Cancer is a global public health issue and poses a major challenge to the standard of medical excellence of all nations. Lung cancer is the second most prevalent cancer worldwide and accounts for the highest cancer-related deaths in both men and women. Globally, 1,796,144 people passed away from this illness in 2020 (1). Small-cell lung cancer (SCLC) and non-small-cell lung cancer (NSCLC) account for the majority of lung cancer cases. Currently, chemotherapy, radiation, and surgery are the main avenues for NSCLC treatment. Given the several investigations on tumor heterogeneity, NSCLC has also been treated with molecular targeted therapy and immunotherapy. However, the 5-year survival rate of NSCLC patients is only 26% (O'Brien and Besse, 2016; Miller et al., 2022). Lung adenocarcinoma is a typical subtype of NSCLC. Despite the discovery of numerous immunological checkpoints and prognostic indicators, the molecular makeup of lung adenocarcinoma remains unclear. There is still an urgent need for more research into treatment targets and prognosis indicators for lung cancer.

MS4A is a new gene family with four transmembrane-spanning domains. Currently, there are at least 16 members in the MS4A family. The MS4A gene family is crucial for cell differentiation, signaling, and cell cycle control (Liang and Tedder, 2001; Eon et al., 2016; Mattiola et al., 2021; Silva-Gomes et al., 2022). Previous research has suggested that members of the MS4A family, including MS4A1, MS4A3, MS4A4A, MS4A6A, MS4A7, MS4A12, and MS4A15, are associated with the onset and progression of cancers; however, the underlying mechanisms remain unknown (Kawabata et al., 2013; Heller et al., 2015; Liang et al., 2020; Pan et al., 2020; Jiang et al., 2021; Mudd et al., 2021; Fang et al., 2022; Luo et al., 2022; Zeng et al., 2022; Zhao et al., 2022). According to the published literature, MS4A2 is strongly associated with the prognosis of lung adenocarcinoma (Ly et al., 2017) and lung cancer brain metastases (Chen et al., 2021). Conversely, MS4A8 is considered to have a role in the morphology and cell development of NSCLC (Kudoh et al., 2020). However, further study of the expression and prognostic significance of the MS4A family in lung adenocarcinoma is needed.

We herein first evaluated the expression of the MS4A family in lung adenocarcinoma and its relationship with clinical patient prognosis and established a prognosis model. We examined the signal route involved by the MS4A family and its connection with the immune microenvironment of lung adenocarcinoma to learn more about the relationship between the pathophysiology of the MS4A family and the disease, which would help establish a theoretical foundation for identifying lung adenocarcinoma prognostic factors and treatment.

## Results

### mRNA expression of the MS4A family in patients with lung adenocarcinoma

**FIGURE 1:**
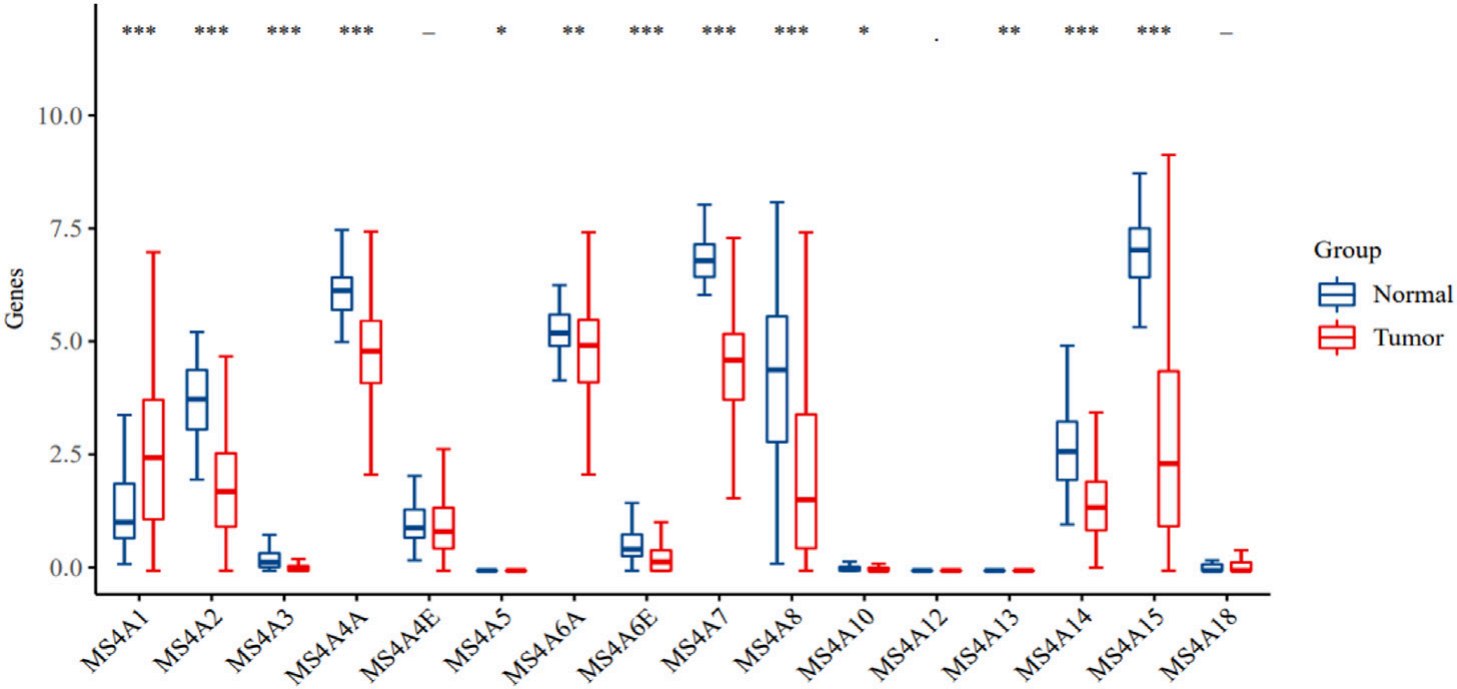
Levels of MS4A family expression in lung adenocarcinoma (TCGA). Blue represents the normal group, while red represents the tumor group. Levels of significance denoted by asterisks are *p < 0.05, **p < 0.01, and ***p < 0.001.

Using the TCGA database, we first looked for MS4A family expression in lung cancer and healthy lung tissues. In lung adenocarcinoma tissues, we discovered that the expression of one MS4A family gene was upregulated, and that of 10 other genes was downregulated (Figure 1). Compared with normal tissues, in lung adenocarcinoma tissues, MS4A2/3/4A/6A/6E/7/8/10/14/15 expression was downregulated, whereas MS4A1 expression was upregulated.

### Mutation landscape of the MS4A family in lung adenocarcinoma

**FIGURE 2:**
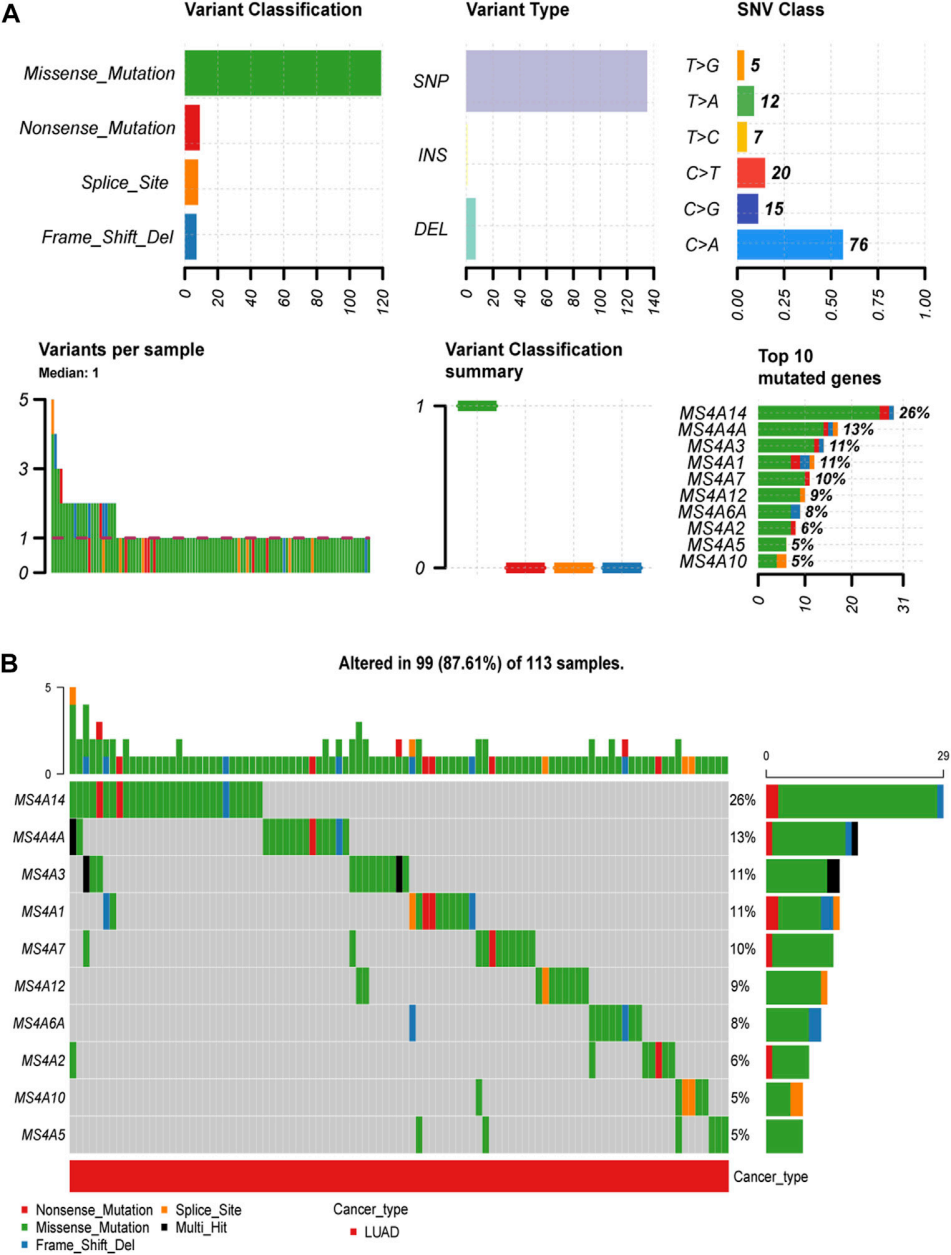
Incidence and classification of MS4A gene mutations in lung adenocarcinoma. **(A)** The types and proportions of mutations in the MS4A family. **(B)** MS4A family mutation frequency in lung adenocarcinoma.

Using the Gene Set Cancer Analysis (GSCA) website, we examined the prevalence of somatic mutations and copy number variants in the MS4A family. Consequently, we discovered that 99/113 lung adenocarcinoma specimens (87.61%) had gene alterations (Figures 2A, B). We discovered that “missense mutations” was the most prevalent variant category, “SNPs” was the most prevalent variant type, and “C > A” was the most prevalent SNV type (see Figure 2A for more details). Among the 18 genes, MS4A14 was the most predominantly mutated gene, followed by MS4A4A, MS4A3, and MS4A1.

### Functional enrichment analysis of the MS4A family

**FIGURE 3:**
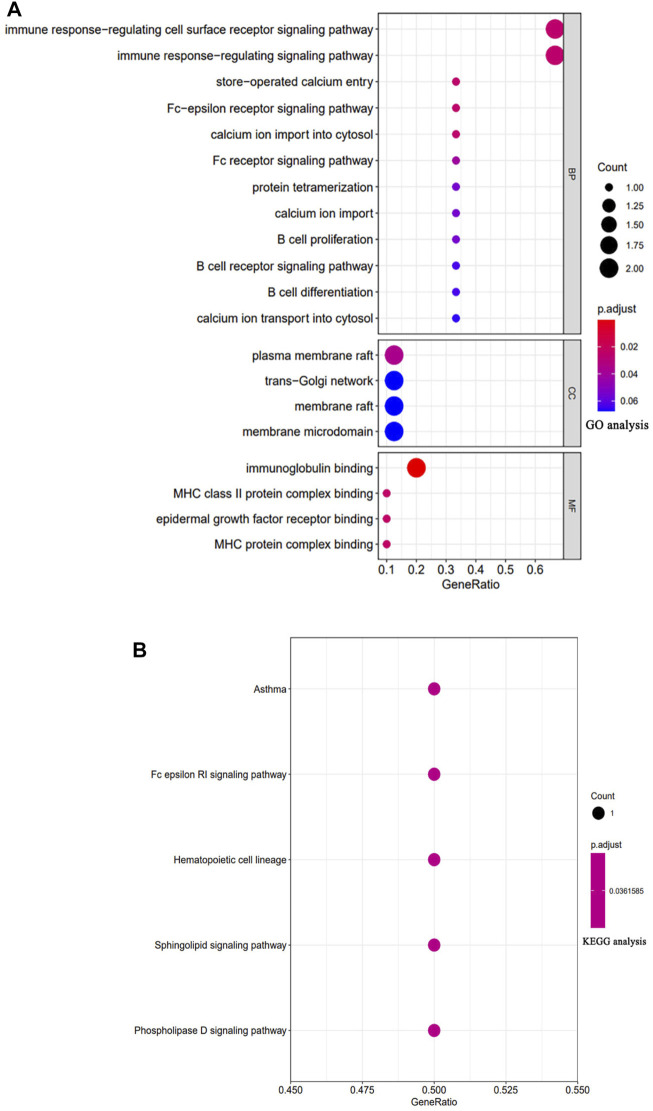
GO and KEGG enrichment analysis results for the MS4A Family. **(A)** The Gene Ontology enrichment analysis. **(B)** The enriched item in the analysis from the Kyoto Encyclopedia of Genes and Genomes. The number of enriched genes is indicated by the size of the circles. MF: molecular function; BP: biological process; and CC: cellular component.

To identify the function of MS4A, we used the R program to evaluate the pathways involving the MS4A family. Through Gene Ontology (GO) analysis, we discovered that the MS4A family was significantly enriched in the immune response regulatory signaling pathway and the immune response regulatory cell surface receptor signaling pathway in the BP category. There was an enrichment in the plasma membrane raft in the CC class. The MF terms were enriched with immunoglobulin binding (Figure 3A). KEGG pathway analysis revealed regulated terms (FDR<0.05), including asthma and FcεRI signaling pathway, hematopoietic cell line, sphingolipids signaling pathway, and phospholipase D signaling pathway (Figure 3B).

### High expression of MS4A2/7/14/15 is beneficial to the survival of lung adenocarcinoma patients

**FIGURE 4:**
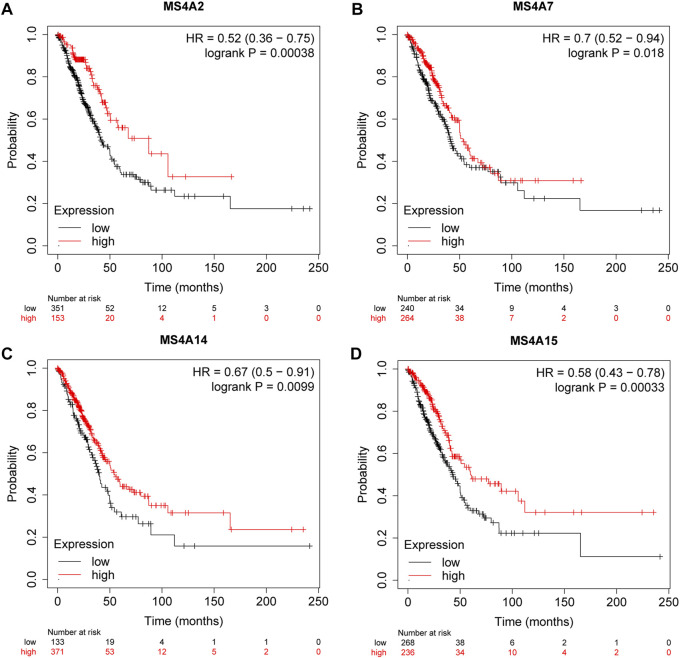
Survival rates for lung adenocarcinoma were associated with the expression of **(A)** MS4A2, **(B)** MS4A7, **(C)** MS4A14, and **(D)** MS4A15 (Kaplan—Meier plotter).

To forecast the predictive value of the MS4A family of genes in lung adenocarcinoma, we used the online database Kaplan–Meier plotter. Then, based on the statistical significance of MS4A’s mRNA expression level, we discovered four genes having a prognostic value. Poorer overall survival was strongly associated with lower expression of MS4A2 (*p* = 0.00038), MS4A7 (*p* = 0.018), MS4A14 (*p* = 0.0099), and MS4A15 (*p* = 0.00033) (Figures 4A–D).

### Construction of an MS4A family prognostic gene model

**FIGURE 5:**
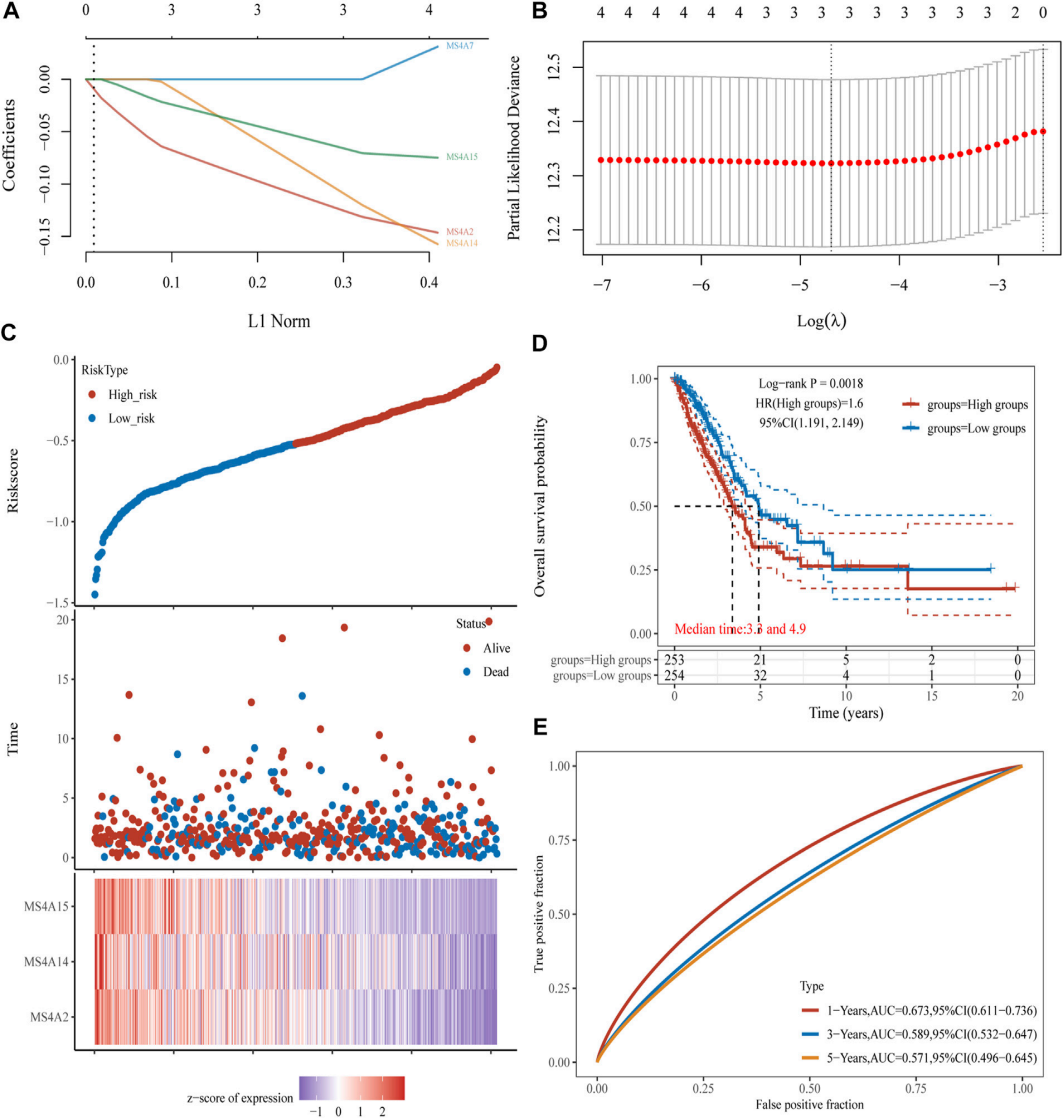
Creating a prognosis model using prognostic genes from the MS4A family. **(A, B)** The partial likelihood deviance on the prognostic genes and the LASSO regression analysis. **(C)** Expression heat map of relevant genes, survival time, and survival status according to risk scores of various samples of lung adenocarcinoma. **(D, E)** The ROC curve of the risk model and the overall survival curve of lung adenocarcinoma patients in the high- and low-risk groups, respectively.

To learn more about the function of prognostic genes in lung adenocarcinoma, four prognostic MS4A genes were used to create a model using LASSO Cox regression analysis. The findings were as follows: risk score = (−0.1232) *MS4A2+(-0.1048) *MS4A14+(-0.0642) *MS4A15 (Figures 5A, B). Lung adenocarcinoma patients were divided into two groups based on the risk score. The risk score distribution, survival status, and expression of these four genes are presented in Figure 5C. The level of gene expression declines as the risk score rises, and consequently, patients have shorter lives. The Kaplan–Meier curve, as seen in Figure 5D, demonstrates that patients with lung adenocarcinoma who have high risk scores have a worse prognosis than those who have low risk scores (median time = 3.3 years vs. 4.9 years, *p* = 0.0018). ROC analysis revealed that the AUC values at 1, 3, and 5 years were 0.673, 0.589, and 0.571, respectively (Figure 5E).

### Construction of a nomogram model

**FIGURE 6:**
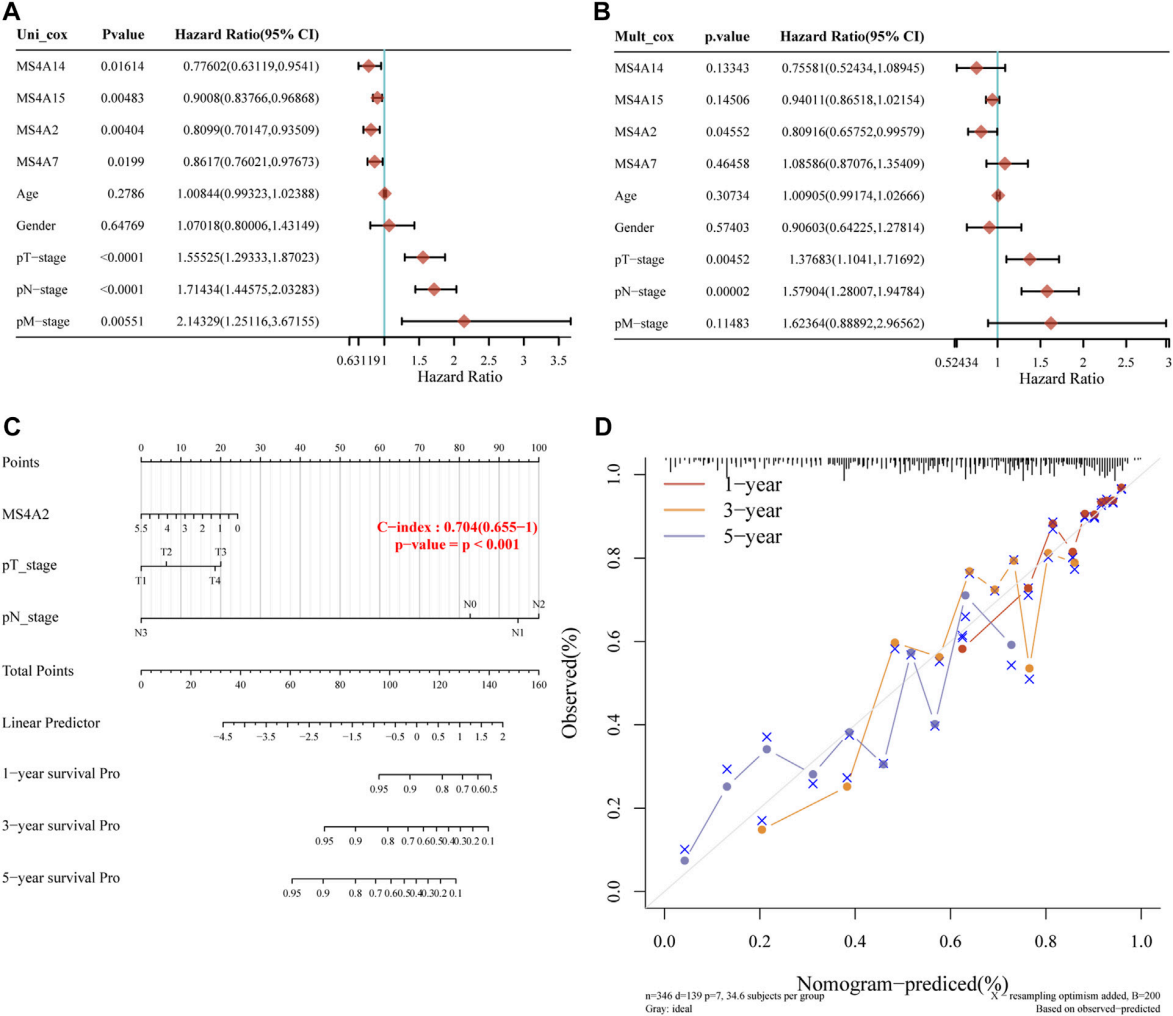
Building a predictive nomogram. **(A, B)** *p*-value and risk ratio of clinical features and prognostic gene expression using univariate and multivariate Cox analyses. **(C, D)** Nomograms can be used to forecast the 1-, 3-, and 5-year overall survival of lung cancer patients. Ideal nomograms are shown by diagonal dotted lines, while 1-, 3-, and 5-year nomograms are represented by red, orange, and blue lines, respectively.

Using the prognostic gene model, we identified the influence of four prognostic genes on the survival rate of lung adenocarcinoma patients. However, several factors affect the prognosis in cancers. To further investigate the effect of prognostic genes and clinical parameters, such as age, sex, and clinical stage, on the overall survival of patients with lung adenocarcinoma, we built a model using nomograms. According to the single-factor Cox regression analysis, MS4A2/7/14/15 are protective factors in lung adenocarcinoma, whereas staging is a risk factor (Figure 6A). Multivariate Cox regression analysis revealed that the prognosis is significantly influenced by clinical stage and MS4A2, suggesting that MS4A2 and the pT (pathologic tumor), pN (pathologic tumor), and pM (prognostic distant metastasis) stages are independent factors affecting the prognosis of lung adenocarcinoma (Figure 6B). A nomogram incorporating variables with appreciable differences compared to the prognosis was created based on the findings of the multivariate analysis. We discovered that the 3- and 5-year overall survival rates could be reasonably predicted when compared to the ideal model of the entire cohort (Figures 6C, D).

### Immune cell infiltration of prognostic genes of the MS4A family in lung adenocarcinoma

**FIGURE 7:**
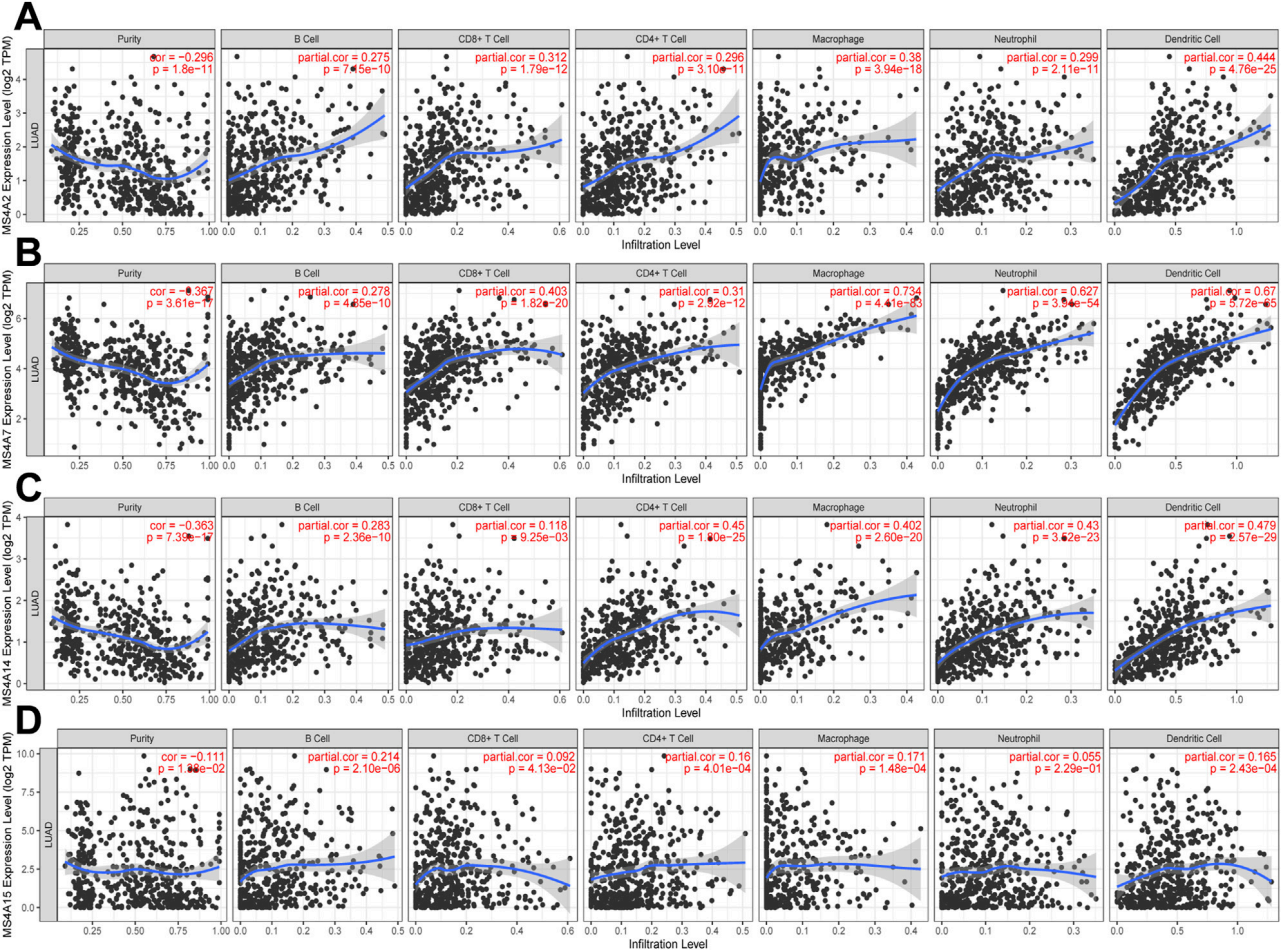
Relationship between immune infiltration and four prognostic MS4As (TIMER). The expression of **(A)** MS4A2, **(B)** MS4A7, **(C)** MS4A14, and **(D)** MS4A15 in lung adenocarcinoma is correlated with the abundance of immune cells.

From GO analysis findings, we learned that the MS4A family is involved in the immune regulation pathway and that the immunological microenvironment is crucial for cancer initiation and development. Using the TIMER database, we discovered a strong association between immune-infiltrating cells and MS4A family prognostic genes in lung adenocarcinoma. We noted a positive correlation between the prognostic genes (MS4A2 and MS4A7) and the quantity of immune-infiltrating cells (B cells, CD4^+^ T cells, CD8^+^ T cells, macrophages, neutrophils, and dendritic cells; Figures 7A, B). The expression of MS4A14 is positively correlated with the infiltration of B cells, CD4+ T cells, macrophages, neutrophils, and dendritic cells, but there is no significant correlation with CD8+ T cells **(** Figure 7C). The expression of MS4A15 is related to B-cell infiltration **(** Figure 7D).

### Validation of prognostic genes in lung adenocarcinoma by real-time quantitative polymerase chain reaction

**FIGURE 8:**
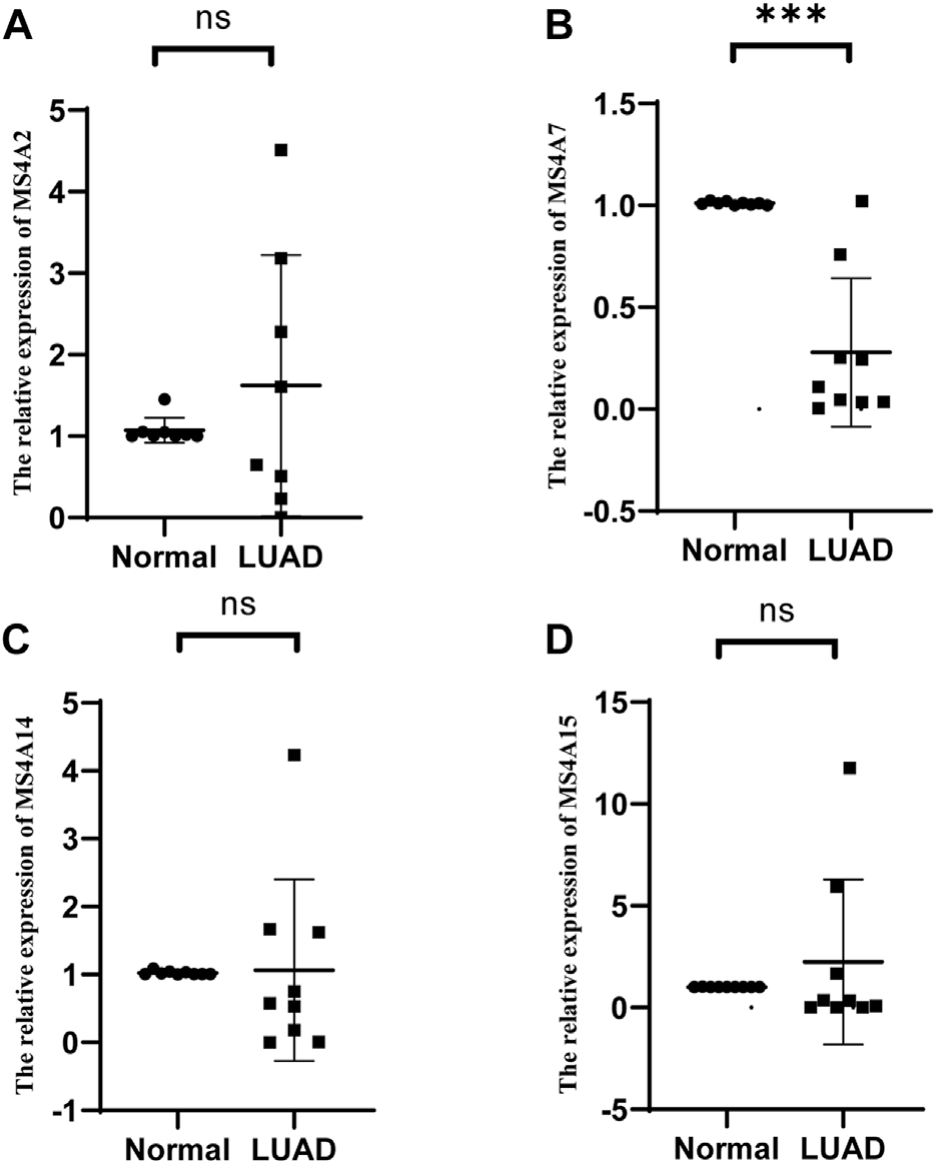
Relative mRNA expression levels of **(A)** MS4A2, **(B)** MS4A7, **(C)** MS4A14, and **(D)** MS4A15 were detected by RT-qPCR in lung adenocarcinoma and healthy lung tissues. Levels of significance denoted by asterisks are *p < 0.05, **p < 0.01, and ***p < 0.001. “ns” represents no statistical significance.

To confirm the expression of prognostic genes of the MS4A family in lung adenocarcinoma, we used real-time quantitative polymerase chain reaction (RT-qPCR) to identify the mRNA expression levels of prognostic genes in lung adenocarcinoma and paired neighboring normal lung tissues. Among the nine pairs of lung adenocarcinoma and neighboring tissues obtained by us, MS4A2 expression in lung adenocarcinoma tissues was considerably downregulated in four pairs and upregulated in five pairs (Figure 8A; Supplementary Figures S2A–I). In one set of samples with upregulated expression, the relative expression of MS4A2 in lung adenocarcinoma tissues was more than 300 times that in normal lung tissues (Supplementary Figures S2A–I), and therefore, it was disregarded. In eight sample pairs, the expression of MS4A7 was dramatically reduced, whereas no significant variation in the expression level was noted in one pair (Figure 8B; Supplementary Figures S3A–I). Compared with normal lung tissues, MS4A14 and MA4A15 expression levels were significantly downregulated in six pairs of lung adenocarcinoma tissues and markedly upregulated in the remaining three pairs (Figures 8C, D; Supplementary Figures S4A–I, S5A–I).

### Discussion

Members of the MS4A family have similar structures and roles. Previous reports indicate that the MS4A protein predominantly interacts with several immunological receptors and controls signaling pathways (Liang and Tedder, 2001; Polyak et al., 2008; Schmieder et al., 2011; Eon et al., 2016; Mattiola et al., 2019; Mattiola et al., 2021). The well-known members of the MS4A family, namely, MS4A1 (CD20), MS4A2 (FcεRIβ), and MS4A3 (HTm4), all have a significant role in cancer initiation and development. However, it is unknown how most members of this family contribute to lung adenocarcinoma.

To determine this, we started by exploring the expression of the MSA4 family in lung adenocarcinoma. Using the TCGA database, we discovered that 11/18 genes in the MS4A family had differential expressions. Based on our research on the prevalence of copy number variation and somatic mutation in the MS4A family, we found that the majority of the MS4A family members had gene mutations in lung adenocarcinoma. Specifically, MS4A14, MS4A4A, MS4A1, and MS4A3 genes had the highest prevalence of mutations. Additionally, we performed a functional enrichment analysis. The findings corroborated prior research by Mattiola and Eon Kuek and revealed that the MS4A family was primarily involved in the receptor signaling pathway on the surface of immune response-regulating cells and the immune response-regulating signaling pathway, which was associated with immunoglobulin binding (Eon et al., 2016; Mattiola et al., 2021). According to their research, the MS4A family is instrumental in humoral immunity, IgE signal transduction, and T-cell proliferation control (Lin et al., 1996; Howie et al., 2009; Kuijpers et al., 2010). Also, the MS4A family contributes to asthma and the FcεRI signaling pathway. Using the STRING website (https://cn.string-db.org/), we created a network map illustrating the interactions between the MS4A protein family and associated proteins. The interactions between the MS4A protein family and associated proteins are depicted in Supplementary Figure S1. The promoter methylation of FCER1G, which is most closely connected to the MS4A protein family, can inhibit the expression of FcεRI in patients with atopic dermatitis (Liang et al., 2012). This indicates that the FCER1G and MS4A protein families are jointly involved in the regulation of the FcεRI pathway. According to prognostic analyses, patients with low expression levels of MS4A2, MS4A7, MS4A14, and MS4A15 had a worse prognosis. The overall survival of patients with lung adenocarcinoma was positively correlated with the expression level of these genes. Then, to better predict the overall survival of lung cancer patients, we built a prognostic model incorporating the four prognostic genes. Using the LASSO Cox regression analysis and prediction nomogram, we found that the model could predict 3- and 5-year overall survival with reasonable accuracy. We discovered a significant positive correlation between prognostic genes and immune-infiltrating cells through immune infiltration analyses, and we also discovered that lung adenocarcinoma patients with low expression of the prognostic MS4A family genes had a poor prognosis. This shows that the downregulation of MS4A prognostic gene expression may have an impact on immune cells’ capacity to proliferate, mature, and kill. However, it is not yet apparent how immune cells will be impacted and at what stage this will appear. Additionally, this is the direction we need to explore next.

Notably, MS4A2, an intensively examined MS4A family member, is a crucial part of high-affinity IgE (Kraft and Kinet, 2007). In agreement with the findings of Ly et al., MS4A2 has low expression in lung adenocarcinoma and is associated with a bad prognosis (Ly et al., 2017). Their findings show that mast cells affect the development of lung cancer and that high MS4A2 expression on stromal mast cells is a positive prognostic sign for the survival of early lung cancer patients. We discovered that as per the prognostic model and nomogram analysis findings, MS4A2 is a protective gene in lung adenocarcinoma and an independent factor impacting prognosis; it is also considerably positively associated with immune-infiltrating cells. Then, using RT-qPCR, we further confirmed MS4A2 expression in lung adenocarcinoma. Notably, only three of the nine sample pairs gathered herein showed reduced expression of MS4A2. In agreement with other research studies, our bioinformatics study showed that the level of MS4A2 was low in lung adenocarcinoma. This could be attributed to the small sample size of this study. Another influencing factor could have been the primers we created because we only chose a small portion of the MS4A2 mRNA, and this may not accurately reflect all functions of MS4A2. As the MS4A family’s first identified ion channel, MS4A2 can function as a calcium channel (Alber et al., 1991; Ishibashi et al., 2001) which is associated with the development of numerous cancers (Gautier et al., 2019). Although this could be a process through which MS4A2 contributes to lung adenocarcinoma, more research is required to precisely determine the underlying pathway and mechanism. The FcεRI receptor is a tetramer complex, one of which, the β subunit, is encoded by MS4A2 (Bitting et al., 2023), suggesting a connection between the MS4A family and the FcεRI signaling pathway, which is consistent with the KEGG enrichment study demonstrating the involvement of the MS4A family in the FcεRI signaling pathway. Previous studies have suggested a link between MS4A2 and the onset of asthma. The mutation in exon 7 E237G may be a risk factor for the development of atopic asthma (Yang et al., 2014); however, the prevalence of asthma is unrelated to the methylation of the MS4A promoter (Ferreira et al., 2010). Further molecular mechanisms need to be studied.

During the study of the MS4A family, it was discovered using PCR amplification that MS4A7 is primarily expressed in B cells and monocytes in hematopoietic cell lines. In addition, MS4A7 is present in non-hematopoietic cell types, such as those found in the colon, thymus, lung, and other organs (Liang and Tedder, 2001; Mattiola et al., 2021). Few reports on MS4A7 in tumors have been published so far, mainly in cases of esophageal and gastric cancer (Sun et al., 2018; Zhou and Wang, 2020). In their research, it has been discovered that the poor prognosis of these two cancers is associated with high MS4A7 expression. Even in lung adenocarcinoma, MS4A7 has been reported to be a predictor of poor survival (Luo et al., 2022). In our study, however, the low expression of MS4A7 in lung adenocarcinoma suggests a bad prognosis. Our findings were further supported by the outcomes of RT-qPCR tests performed on lung cancer tissues and healthy lung tissues. In addition, the immune infiltration analysis revealed a strong correlation between MS4A7 and immune cells, particularly macrophages and dendritic cells, in lung adenocarcinoma. In contrast to our prediction results, which may be attributable to the various databases and analysis techniques used by us, Luo et al. (2022) reported MS4A7 as a predictor of poor lung adenocarcinoma; however, they did not analyze the expression level of MS4A7 in lung adenocarcinoma. The bad prognosis associated with high MS4A7 expression in gastric and esophageal cancers may be associated with the ability of MS4A7 to control tumor growth in lung adenocarcinoma through various other mechanisms; however, the specific mechanism of MS4A7 in lung adenocarcinoma remains to be confirmed.

Recent investigations have demonstrated that MS4A14 is highly expressed in renal clear cell carcinoma and that individuals with high MS4A14 expression have lower overall survival rates (Li et al., 2022). Conversely, patients with low expression of MSA14 in lung adenocarcinoma reportedly have a bad prognosis. The prognosis model has enabled us to determine that MS4A14 is a lung adenocarcinoma protective factor that is favorably correlated with patient survival time. However, the biological functions of MS4A14 remain poorly understood, and more research is required to determine how MS4A14 affects lung adenocarcinoma.

The MS4A family is closely related to calcium channels (Eon et al., 2016; Mattiola et al., 2021), and MS4A15, which controls the level of calcium ions to coordinate lipid remodeling and prevent iron death, has recently been shown to be present in the endoplasmic reticulum (Xin et al., 2022). According to several studies, MS4A15 is upregulated in ovarian cancer and can encourage the proliferation of ovarian cancer cells both *in vivo* and *in vitro* (Fang et al., 2022).

In our experiment, we found the expression of MS4A15 in lung cancer to be downregulated relative to that in normal lung tissues, indicating a negative prognosis for patients. Since MS4A15 is found in the endoplasmic reticulum, it possibly controls iron death and the structure and function of the mitochondria to influence the onset and progression of malignancies.

This study has some limitations. *In vivo* and *in vitro* tests are primarily lacking, and the mechanism behind the involvement of the MS4A family in lung adenocarcinoma remains to be identified.

In summary, we thoroughly examined the expression and prognosis of the MS4A family in lung adenocarcinoma and identified four MS4A family genes with prognostic value. Additionally, we found a strong association between prognostic genes and immune infiltration, and prognostic genes may influence lung adenocarcinoma development via calcium channels.

## Methods

### Identification of differentially expressed MS4As

RNA-sequencing expression (level 3) profiles and associated clinical data for lung adenocarcinoma were retrieved from the TCGA database (https://portal.gdc.com; Supplementary Table S1) (Zhou et al., 2020; Jin et al., 2021). R version 4.0.3 was used to apply all analysis techniques and packages.

### Mutation analysis of the MS4A family

We used the online database GSCA (http://bioinfo.life.hust.edu.cn/GSCA/#/mutation) to study the gene mutation landscape of the MS4A family in lung adenocarcinoma, and we used the TCGA database to gather SNV data from 113 lung cancer samples for analysis. Seven different mutation types were examined: Missense_ Mutation, Nonsense_ Mutation, Frame_ Shift_ Ins, Splice_ Site, Frame_ Shift_ Del, In_ Frame_ Del, and In_ Frame_ Ins. These mutations were called *detrimental mutations*.

### Functional enrichment analysis

Following the collection and arrangement of data from the TCGA database, functional enrichment studies were carried out using the tools clusterProfiler v4 2.0 and org.Hs.eg.db v3.14.0, and bubble charts were produced. If there were numerous notable entries among them, the top 20 were automatically shown in the figure.

### Prognostic analysis of differentially expressed genes

To assess the predictive significance of the mRNA expression of MS4A family members in patients with lung adenocarcinoma, the Kaplan–Meier plotter (http://kmplot.com/analysis/) was used. This plotter can help compare the 30 K gene (mRNA, miRNA, and protein) expression and survival rate associated with 21 tumor types, including breast cancer, ovarian cancer, lung cancer, and gastric cancer. The primary goal is to locate and validate biomarkers using a meta-analysis. The data primarily come from TCGA, EGA, and GEO (Nagy et al., 2021). On the basis of the median expression (high expression and low expression), patient samples were split into two groups in the Kaplan–Meier plotter, and their outcomes were assessed using the Kaplan–Meier survival map, risk ratio (HR) of 95% confidence interval (CI), and log-rank *p*-value. A *p*-value of 0.05 or lower was considered to indicate a statistically significant difference.

### Construction of four gene prognostic models

Lung adenocarcinoma RNA-sequencing expression (level 3) profiles and associated clinical data (Supplementary Table S2) were downloaded (https://portal.gdc.com) from the TCGA dataset. Samples with clinical information were retained while converting counts data to TPM and normalizing the data log2 (TPM+1). Consequently, a total of 516 samples were collected for analysis. The survival differences between healthy individuals and patients with lung adenocarcinoma were tested using log-rank tests, and the predictive model’s accuracy was evaluated using timeROC (v0.4) analysis (Ji and Xue, 2020; Zhang et al., 2020; Xu et al., 2021). R (foundation for statistical computing 2020) version 4.0.3 was used to implement all analysis techniques and R packages. A *p*-value of 0.05 was considered to indicate statistical significance.

### Construction of a nomogram

The lung adenocarcinoma RNA-sequencing expression (level 3) profiles and the associated clinical data (Supplementary Table S3) were downloaded from the TCGA dataset (https://portal.gdc.com). Univariate and multivariate Cox regression analyses were used to choose the appropriate phrases to construct a nomogram (Liu et al., 2020). Through the “forestplot” R package, the forest plot was used to display the *p*-value, HR, and 95% CI of each variable (Jeong et al., 2020; Xiong et al., 2020). A nomogram based on the outcomes of the multivariate Cox proportional hazards analysis was created to forecast the overall recurrence over the next 5 years.

### Immune infiltration analysis

The TIMER web server (https://cistrome.shinyapps.io/timer/) is a comprehensive resource for the systematic investigation of immune infiltrates in various cancer types (Li et al., 2016; Li et al., 2017). Six immunological infiltrates (B cells, CD4^+^ T cells, CD8^+^ T cells, neutrophils, macrophages, and dendritic cells) can be estimated by TIMER. In this study, the relationship between prognostic genes and immune-infiltrating cells was examined using the “Gene Module.

### RNA extraction and RT-qPCR

Nine patients with lung adenocarcinoma had their normal and cancerous lung tissues removed at the First Hospital of Jilin University, and total RNA was isolated using TRIzol (GenStar, China). We used a Uni kit (TransGen, China) to reverse transcribe RNA. Then, we performed RT-qPCR to determine the degree of cDNA expression using 2 × RealStar Green Fast Mixture (GenStar) as an internal control. The geometric mean of housekeeping gene GAPDH was used as an internal control to normalize the variability in expression levels. We used 2^−ΔΔCT^ to determine the relative gene expression level, and GraphPad 8.0 was used to display the results. The difference between the two groups was compared. The data conformed to the normal distribution using the *t*-test, and the data did not conform to the normal distribution using the Wilcoxon test. The data were expressed as mean ± SD, with *p* < 0.05 indicating a significant difference. Supplementary Table S4 enlists the primer sequences used for RT-qPCR. The relative expression of prognostic gene mRNA in lung adenocarcinoma and normal lung tissues is presented in Supplementary Table S5. Each patient provided written informed consent and agreed to participate in the trial. The research methodologies followed the guidelines outlined in the Helsinki Declaration. The research protocol was approved by the First Hospital of Jilin University Ethics Committee.

## Data Availability

The original contributions presented in the study are included in the article/Supplementary Material; further inquiries can be directed to the corresponding author.
