# Supplementary figures and images for: Role of the membrane-spanning 4A gene family in lung adenocarcinoma
Source: Front Genet. 2023 Jul 18;14:1162787. doi: 10.3389/fgene.2023.1162787 (PMC10390740; doi:10.3389/fgene.2023.1162787)

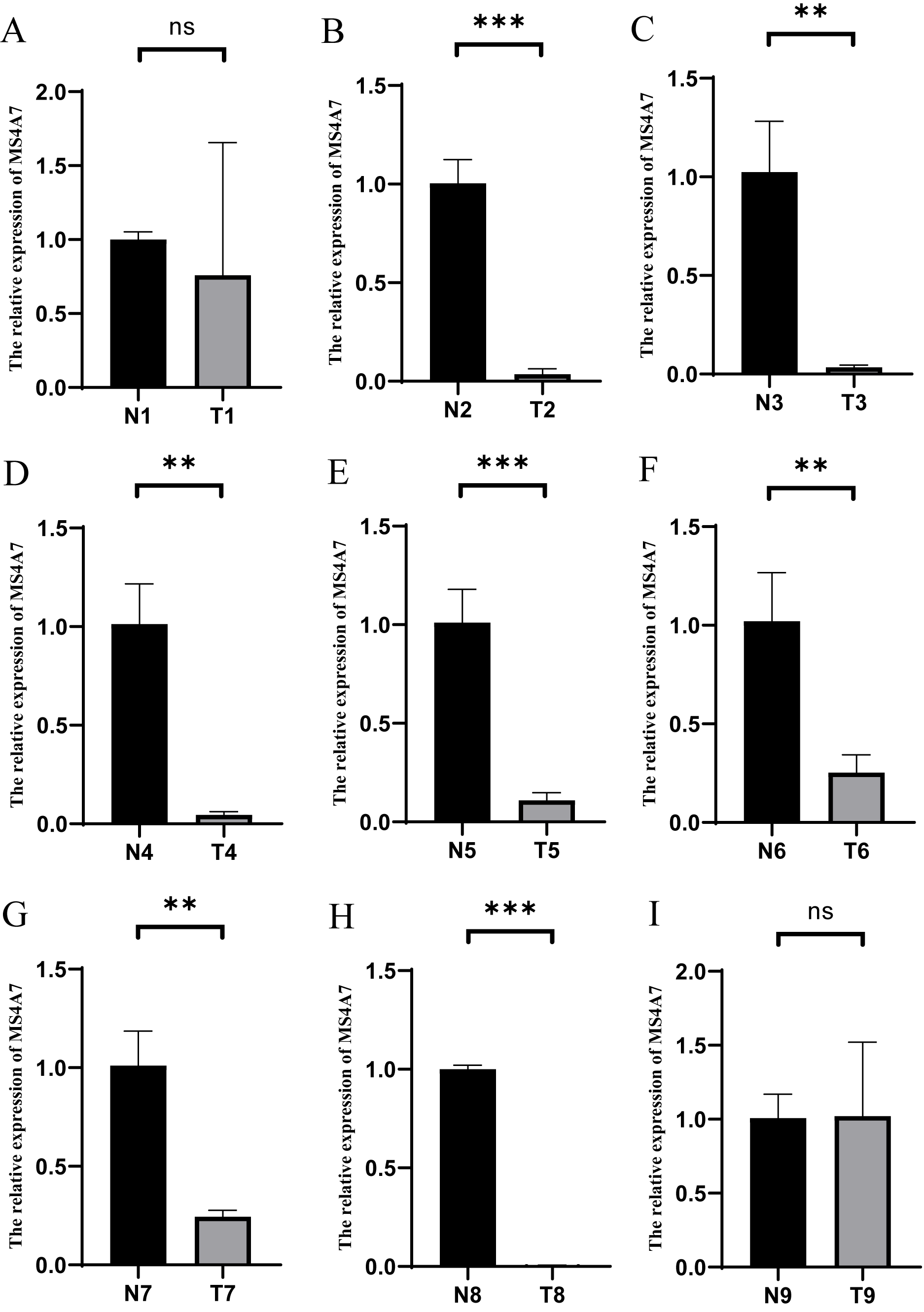

Supplement: Supplementary file 3 [file Image3.TIF]

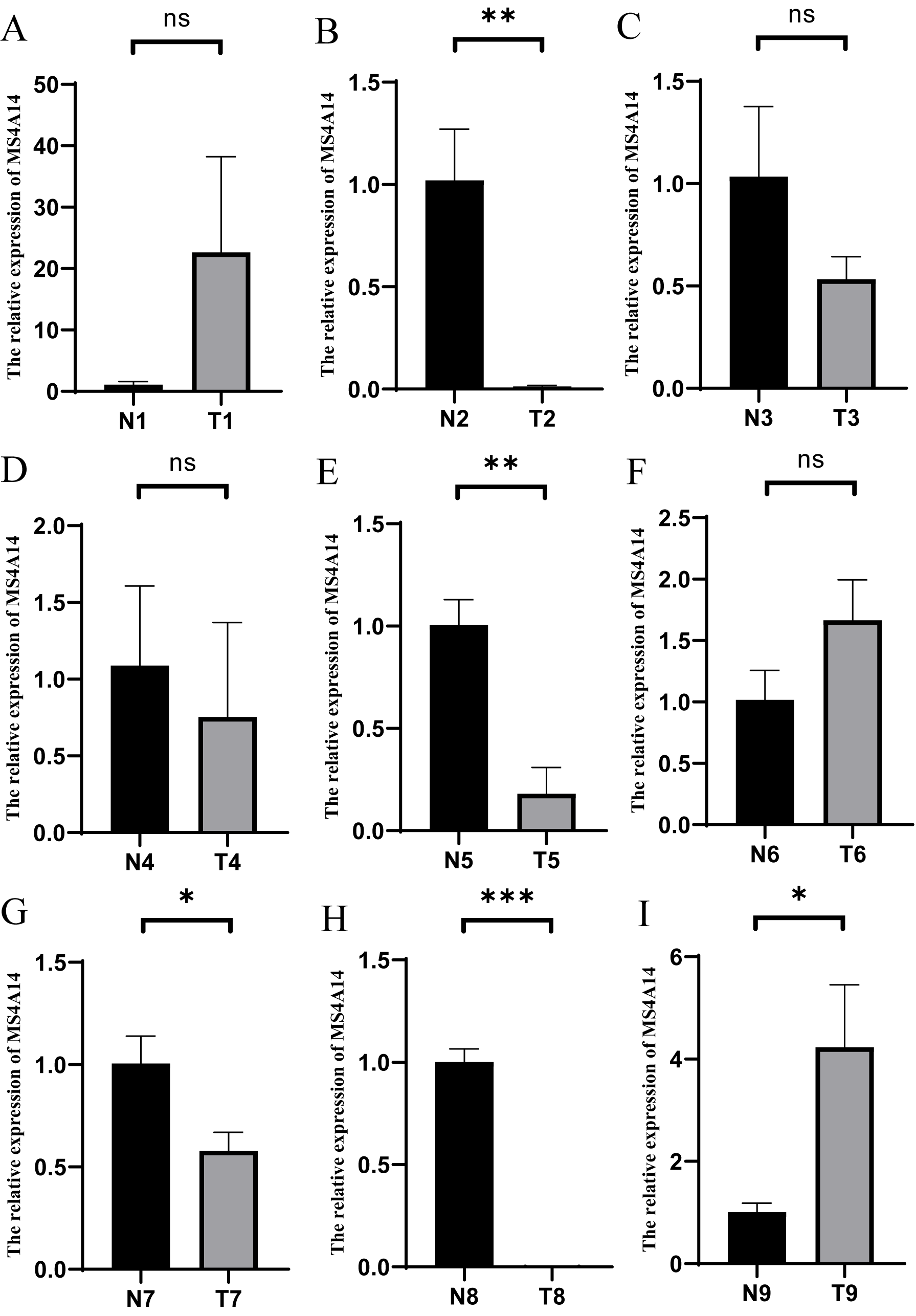

Supplement: Supplementary file 4 [file Image4.TIF]

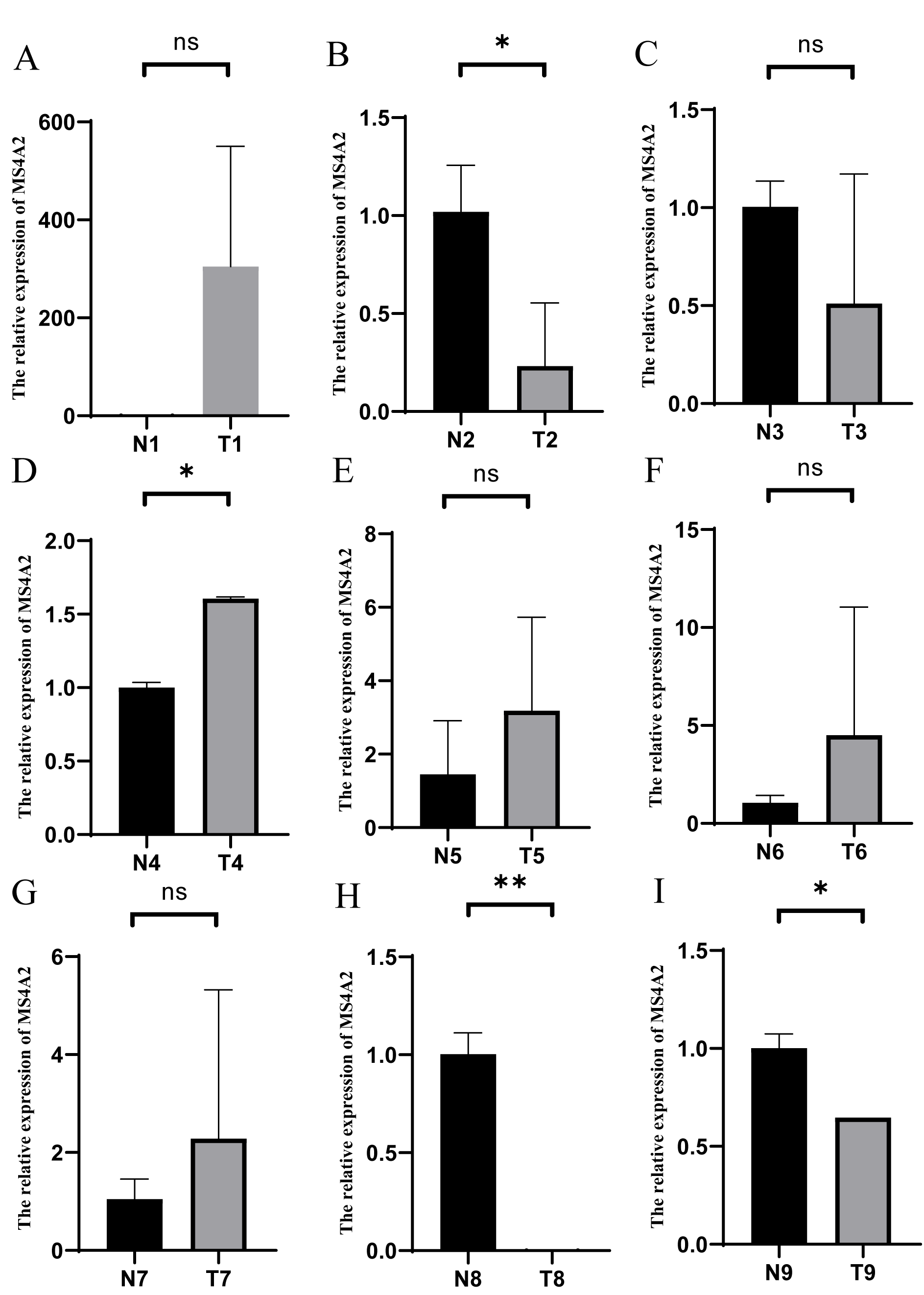

Supplement: Supplementary file 5 [file Image2.TIF]

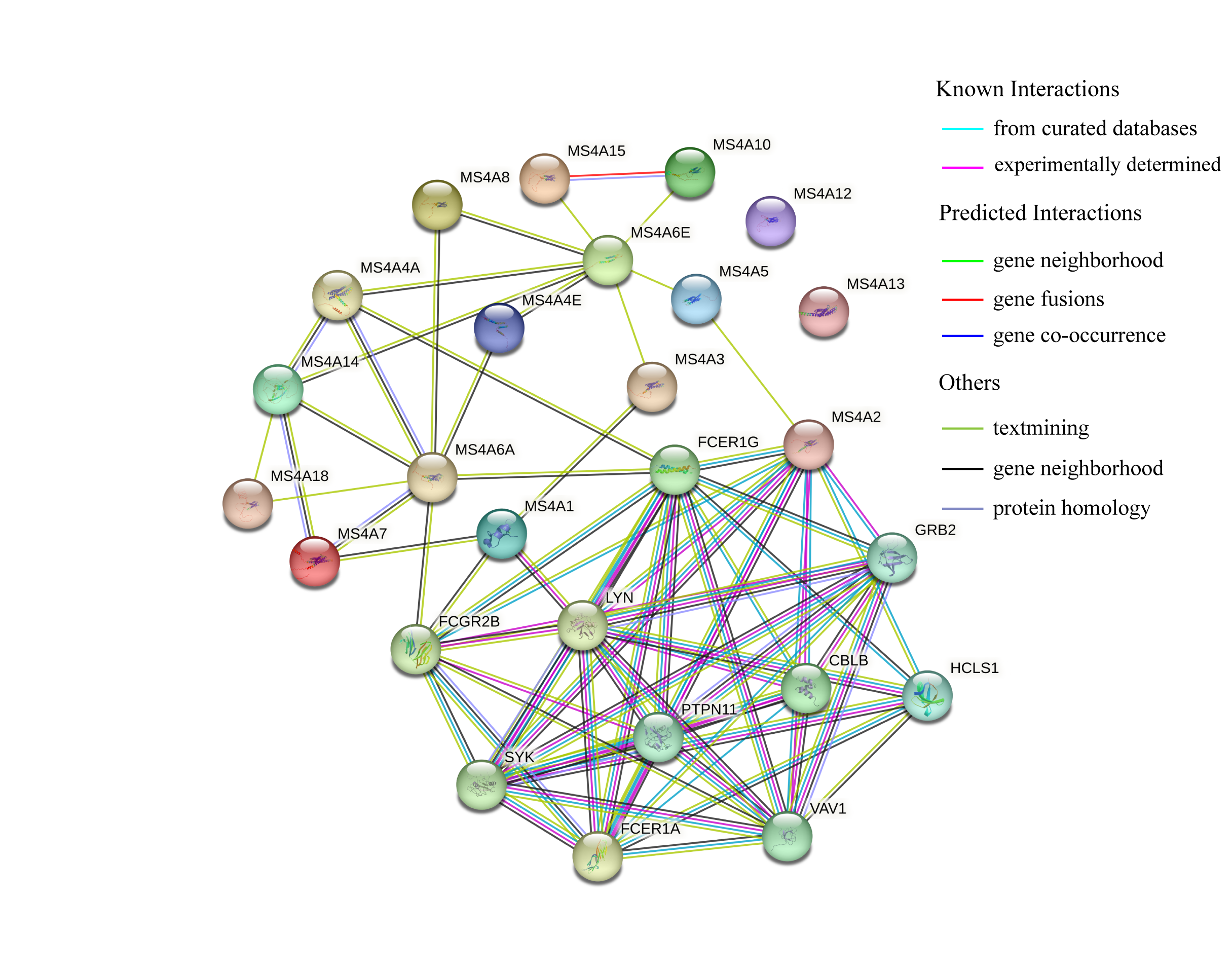

Supplement: Supplementary file 6 [file Image1.TIF]

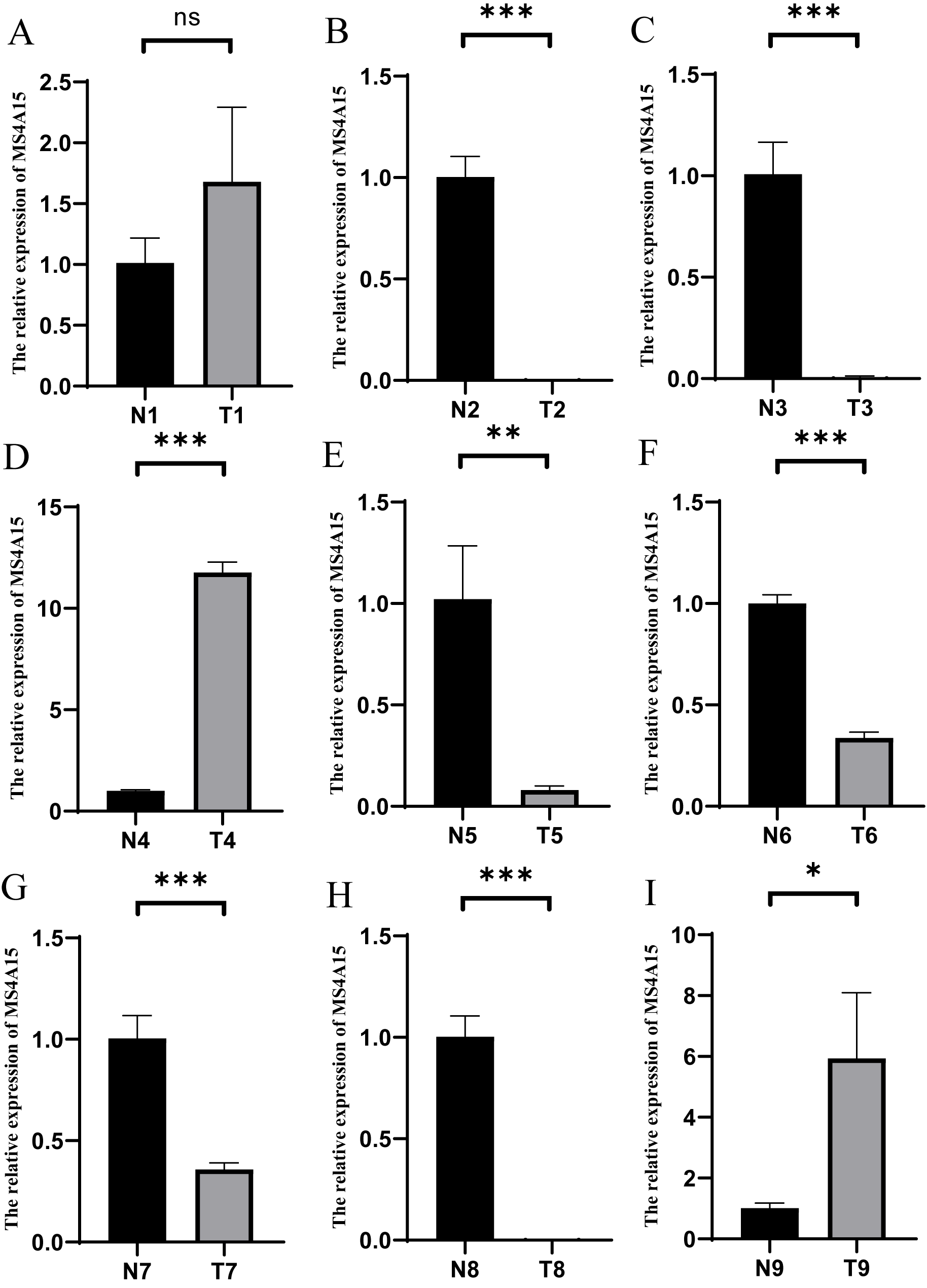

Supplement: Supplementary file 9 [file Image5.TIF]
